# Bell-state measurement exceeding 50% success probability with linear optics

**DOI:** 10.1126/sciadv.adf4080

**Published:** 2023-08-09

**Authors:** Matthias J. Bayerbach, Simone E. D’Aurelio, Peter van Loock, Stefanie Barz

**Affiliations:** ^1^ Institute for Functional Matter and Quantum Technologies, University of Stuttgart, 70569 Stuttgart, Germany.; ^2^ Center for Integrated Quantum Science and Technology (IQST), University of Stuttgart, 70569 Stuttgart, Germany.; ^3^ Johannes-Gutenberg University of Mainz, Institute of Physics, Staudingerweg 7, 55128 Mainz, Germany.

## Abstract

Bell-state projections serve as a fundamental basis for most quantum communication and computing protocols today. However, with current Bell-state measurement schemes based on linear optics, only two of four Bell states can be identified, which means that the maximum success probability of this vital step cannot exceed 50%. Here, we experimentally demonstrate a scheme that amends the original measurement with additional modes in the form of ancillary photons, which leads to a more complex measurement pattern, and ultimately a higher success probability of 62.5%. Experimentally, we achieve a success probability of (57.9 ± 1.4)%, a substantial improvement over the conventional scheme. With the possibility of extending the protocol to a larger number of ancillary photons, our work paves the way toward more efficient realizations of quantum technologies based on Bell-state measurements.

## Teaser

A linear-optical measurement scheme can distinguish Bell states with more than 50% success probability.

## INTRODUCTION

One of the most iconic applications of a Bell-state measurement (BSM) is quantum teleportation, where an arbitrary and unknown quantum state is teleported from one party to another by sharing entanglement between the parties and subsequently performing a BSM on the state to be teleported and one-half of the entangled pair (*1*–*8*). A BSM here means a projection onto a maximally entangled basis, the Bell basis.

Quantum teleportation and, in turn, BSMs themselves now serve as important primitives for many other protocols underlying quantum technologies. In particular, in quantum communication, BSMs facilitate entanglement swapping and thereby the implementation of quantum repeaters. Furthermore, BSMs enable the realization of measurement-device-independent quantum communication (*9*–*16*). In quantum computing, BSMs have an important role in photonic quantum computing, particularly in measurement- and fusion-based approaches (*7*–*22*), where BSMs are an integral part of the generation of resource states for photonic quantum computing and in fusing small-scale units to large resource states for the realization of quantum error correction (*22*, *23*). Moreover, BSMs can be used to link distant stationary quantum computers or quantum registers via optical channels, thus creating a quantum internet (*24*–*29*).

The standard approach to realizing an optical BSM is letting two entangled photons impinge on two inputs of a balanced beam splitter and measuring the resulting output patterns and statistics (*3*, *30*). The simplicity of such a linear-optical approach comes at the cost of being able to identify only two of four possible Bell states. This means that in 50% of all cases, the obtained results are ambiguous (*31*). This limitation directly affects any optical quantum technology that relies on successful projection onto the Bell basis.

In atomic systems, complete BSMs have been performed (*5*, *6*, *32*, *33*). However, these require intricate experimental setups that are challenging to scale up. Complete BSMs on spin qubits are possible in solid-state systems (*34*), but thermal effects typically prevent operations at room temperature. In general, nonoptical approaches suffer from limited intrinsic clock rates of the order of megahertz. Only the photonics platform offers, in principle, high processing clock rates at room temperature. Optical, continuous-variable Bell measurements and quantum teleportation (*8*) are deterministic and well scalable; however, a practical mechanism for loss detection and general quantum error correction is lacking.

For the photonic qubit approach, both scalability and fault tolerance in quantum communication and computing heavily depend on the BSM efficiencies. In this case, the 50% limit has been overcome in proof-of-principle experiments by exploiting hyperentanglement (*35*–*37*) and incorporating nonlinear elements (*4*, *5*, *33*, *38*). A third approach that has been suggested in theory is based on adding additional ancillary photons to a linear-optics setup (*39*, *40*) and using photon-number resolving detectors, offering clear advantages regarding lower experimental complexity and higher scalability.

In this work, we demonstrate such a linear-optical BSM scheme enhanced by ancillary photons by adapting and implementing a scheme proposed in (*40*). In our experiments, we surpass the fundamental limit of 50% demonstrating an experimental success probability of 57.9%. The maximum theoretical value for our approach is five-eighth (*40*, *41*). We achieve this enhanced success probability using additional single photons, expanding of the linear-optical circuits and realizing pseudo-photon-number resolution using 48 single-photon detectors. Such an improved BSM efficiency can have a substantial impact on practical quantum technologies, and examples will be discussed. In particular, these primitives are easily compatible with optical quantum computing and quantum networks, and our results thus present a key step toward highly efficient Bell-state measurements for quantum technology.

## RESULTS

### Theoretical background

The Bell states are maximally entangled two qubit states and are given by

$$|\Psi^{\pm }\rangle =\frac{1}{\sqrt{2}}(|01\rangle_{ab}\pm |10\rangle_{ab})=\frac{1}{\sqrt{2}}(a_{0}^{†}b_{1}^{†}\pm a_{1}^{†}b_{0}^{†})|vac\rangle$$ (1)

$$|\Phi^{\pm }\rangle =\frac{1}{\sqrt{2}}(|00\rangle_{ab}\pm |11\rangle_{ab})=\frac{1}{\sqrt{2}}(a_{0}^{†}b_{0}^{†}\pm a_{1}^{†}b_{1}^{†})|vac\rangle$$ (2)

Here, ∣0⟩ and |1⟩ denote the logical states of the two qubits *a* and *b*, respectively. We can rewrite these terms in the form of the creation operators $a_{0,1}^{†}$ and $b_{0,1}^{†}$, which denote the creation of a photon in the spatial mode *a*, *b* in the state 0 or 1 (for instance, encoded in two polarization modes, ∣0⟩ ≡ ∣*H*⟩ and ∣1⟩ ≡ ∣*V*⟩). The set of Bell states forms a basis of the two-qubit Hilbert space, the Bell basis.

**Fig. 1.**
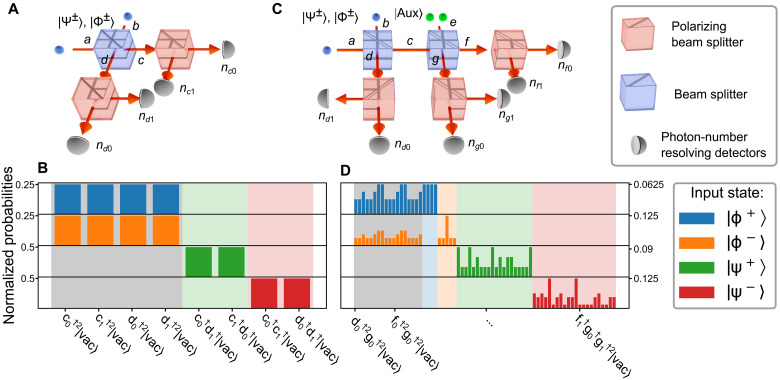
Schematics of the standard and enhanced BSM and the corresponding detection-probability distributions. (**A**) Schematic of a standard BSM and (**C**) a BSM enhanced with an auxiliary state $|\mathrm{Aux}\rangle =\frac{1}{2}(e_{0}^{†}e_{0}^{†}+e_{1}^{†}e_{1}^{†})|vac\rangle$, as well as their respective input-dependent detection-probability distributions (**B** and **D**). Different input states are colored, and identifiable detection patterns are highlighted, while ambiguous ones are accentuated in gray. For the enhanced BSM, the setup is extended by a second beam splitter. Upon comparing the detection probability distributions, the advantage of the enhanced BSM becomes clear, as the two states ∣Φ^±^⟩ are now in 25% of the cases identifiable. Each set of output states is normalized so that the total probability is 1.

In an ideal BSM, when inputting one of the four Bell states, we would identify that specific Bell state with 100% probability. For optical qubits, BSMs can be realized with a setup consisting of a beam splitter (*42*). The Bell state impinging on a balanced beam splitter (see Fig. 1) transforms into the following output states

$$|\Psi^{+}\rangle \to \frac{1}{\sqrt{2}}(c_{0}^{†}d_{1}^{†}+c_{1}^{†}d_{0}^{†})|vac\rangle$$ (3)

$$|\Psi^{-}\rangle \to \frac{1}{\sqrt{2}}(c_{0}^{†}c_{1}^{†}-d_{0}^{†}d_{1}^{†})|vac\rangle$$ (4)

$$|\Phi^{\pm }\rangle \to \frac{1}{2\sqrt{2}}(c_{0}^{†}c_{0}^{†}\pm c_{1}^{†}c_{1}^{†}+d_{0}^{†}d_{0}^{†}\pm d_{1}^{†}d_{1}^{†})|vac\rangle$$ (5)

where *c* and *d* are the output modes of the beam splitter (see Fig. 1A). Measuring the output according to the individual spatial modes and qubit states {0,1} results in a distinctive photon pattern for the states ∣Ψ^±^⟩. However, we see that the states ∣Φ^±^⟩ cannot be uniquely identified (Fig. 1B). Note that linear-optical setups can be designed to distinguish between different sets of states, but the total success probability of the unambiguous output patterns combined never exceeds one-half, imposing the 50% limit (*31*).

We will now show how this probability can be improved using additional ancillary photons in the state [see also (*40*)]

$$|\mathrm{Aux}\rangle =\frac{1}{2}(e_{0}^{†}e_{0}^{†}+e_{1}^{†}e_{1}^{†})|vac\rangle$$ (6)

We use another beam splitter (Fig. 1C) and send the ancillary state into one of the inputs of this second beam splitter (mode *e*). We send one output mode *c* from the first beam splitter to the other input of this second beam splitter. After this second beam splitter, we obtain an output state that consists of four photons distributed over the modes *d*, *f*, and *g* (see Fig. 1C). We observe a distinct photon-number distribution for each Bell state as shown in Fig. 1D.

As before in the standard scheme, we can still distinguish the states ∣Ψ^±^⟩ with 100% probability. However, we get now in addition a subset of unique signatures for the states ∣Φ^+^⟩ and ∣Φ^−^⟩ in 25% of the cases (see Fig. 1D). This means that the overall probability to correctly identify a Bell state for this scheme is *p*_c_ = 62.5% (*40*). At the cost of more ancillary photons, this scheme can be expanded to reach success probabilities close to unity (*39*, *40*). This protocol can be summarized as a black box that takes one of the Bell states as input and outputs a label that can be either one of the four Bell states or an inconclusive result.

We now define a number of parameters that will allow us to quantify the success of the BSM. First, we assume that we send in a particular Bell state. In general, a measurement outcome can either be unambiguous or ambiguous, meaning that the pattern does or does not allow for the identification of a specific Bell state. Within the unambiguous results, we can then have two cases: either the BSM outputs the correct label, or it does not.

Thus, the first quantity is the probability of an unambiguous and correct result *p*_c_: the probability to obtain an unambiguous and correct result given a certain Bell state as input and a measurement outcome *m*, averaged over all possible Bell states

$$p_{c,\mathrm{total}}=\frac{1}{4}\sum_{|\psi \rangle \in \{|\Psi^{\pm }\rangle ,|\Phi^{\pm }\rangle \}}P\left(m=\psi ||\psi \rangle \right)$$ (7)

This probability can reach up to 62.5% in the case of our enhanced scheme.

Because of experimental imperfections, the scheme might output an unambiguous result, which is not consistent with the input state. The probability for unambiguous and false measurement *p*_f_ is given by

$$p_{f,\mathrm{total}}=\frac{1}{4}\sum_{|\psi \rangle \in \{|\Psi^{\pm }\rangle ,|\Phi^{\pm }\rangle \}}P\left(m\neq \psi ||\psi \rangle \right)$$ (8)

and we have *p*_c_ + *p*_f_ = 1 − *p*_amb_, where *p*_amb_ is the probability for an ambiguous measurement.

Knowing *p*_c_ and *p*_f_ allows us to derive the measurement discrimination fidelity, defined as (*43*)

$$\mathrm{MDF}=\frac{p_{c}}{p_{c}+p_{f}}$$ (9)

The MDF denotes the percentage of all correct labels in the subset of all unambiguous results and lies between zero (all labels are incorrect) and one (all labels are correct).

Last, to analyze the complete output statistics, we use the total variation distance *D* (*44*):

$$D=\sum \frac{|f_{i}-q_{i}|}{2}$$ (10)

where *q_i_* is the expected theoretical probability, while *f_i_* is the measured relative probability for the *i*th four-photon output pattern.

### Experimental design

**Fig. 2.**
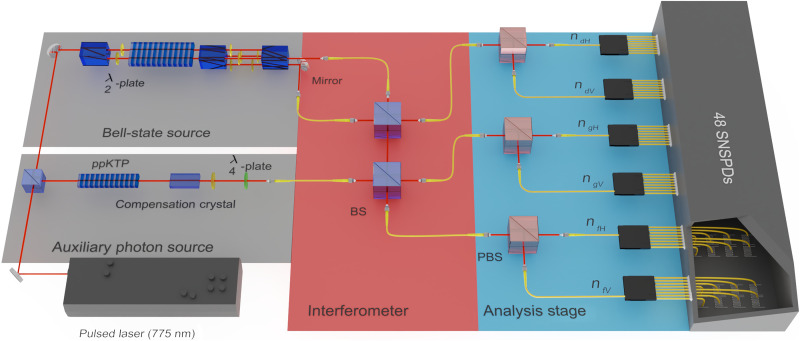
Schematic representation of an SPDC-based enhanced BSM. The setup consists of a Bell-state and auxiliary state source (left, gray area), an enhanced BSM interferometer (middle, red area), and an analysis stage (right, blue area). The auxiliary source uses a ppKTP for photon pair generation, a compensation crystal to counteract the resulting temporal walk-off, and waveplates to set the correct state. For the Bell-state source, a ppKTP crystal is placed inside a Mach-Zehnder–like interferometer. The generated Bell-state photons enter the upper beam splitter. Afterwards, the photons from one output are sent to the lower beam splitter, together with the auxiliary state. The three output modes are then split by PBSs resulting in six spatial modes, which are each again split into eight for detection purposes. This leaves 48 detection modes each of which are connected to a superconducting nanowire detector. Registered detection events are analyzed by a timetagger system, and the number of photons in each spatial mode is computed.

Whereas the original proposal was based on information being encoded in the path of single photons (*40*), in our implementation, we use polarization as a degree of freedom: $a_{0}^{†}:=a_{H}^{†}$ and $a_{1}^{†}:=a_{V}^{†}$. Note that the scheme can be adapted to arbitrary encodings of photonic qubits. We generate both photonic Bell states and the ancillary states using parametric down-conversion in periodically poled potassium titanyl phosphate (ppKTP) crystals (see Fig. 2). For the generation of the different Bell states (see Eqs. 1 and 2), we use a collinear arrangement that generates polarization-entangled states using a Mach-Zehnder-type interferometer (*45*). We characterize the quality of the Bell states by performing correlation measurements, both in the basis {*H*, *V*} and the basis {+,−} with $+=1/\sqrt{2}(a_{H}^{†}+a_{V}^{†})|vac$⟩ and $-=1/\sqrt{2}(a_{H}^{†}-a_{V}^{†})|vac$⟩, obtaining visibilities of *V*_*H*/*V*_ = 0.975 ± 0.003 and *V*_+/−_ = 0.954 ± 0.004. The auxiliary state is obtained with a second collinear setup. The photons are generated from the crystal in the product state $e_{H}^{†}e_{V}^{†}|vac\rangle$ and, with the use of a half-waveplate and a quarter-waveplate at respective angles θ_HWP_ = 22.5° and θ_QWP_ = 45°, the final state becomes $\mathrm{Aux}=\frac{1}{2}(e_{H}^{†}e_{H}^{†}+e_{V}^{†}e_{V}^{†})|vac$⟩. We measured the visibility of the auxiliary state in the basis {*H*, *V*}, achieving a value of *V*_*H*/*V*_ = 0.9899 ± 0.0001. The uncertainties are then estimated by assuming Poissonian distribution of the measured coincidence counts.

The apparatus for the BSM is composed of two balanced beam splitters with the outputs being coupled into single-mode fibers. After passing through the beam splitters, the photons are routed to an analysis stage, which allows performing polarization measurements using polarizing beam splitters (PBSs). The six output modes of this stage will be labeled as *d*_*H*, _*d*_*V*_, *f*_*H*, _*f*_*V*_, *g*_*H*, _and *g*_*V*_ (Fig. 2). The final state is defined in a Hilbert space described by the following set of basis vectors

$$\left\{|n_{dH}n_{dV}n_{fH}n_{fV}n_{gH}n_{gV}\rangle |\sum_{i}\;n_{i}=4\right\}$$ (11)

where *n* indicates the photon number of each particular mode. Each of these modes is further split up into eight spatial modes using a fiber-based 1 × 8 splitter, allowing pseudo-photon-number resolution. The photons are detected using superconducting nanowire single-photon detectors (SNSPDs) with a detection efficiency of on average 88.6%.

To compare both BSM methods, two sets of measurements are performed for each Bell state {∣Ψ^±^⟩, ∣Φ^±^⟩}. The first one is the standard approach, in which the photons from the Bell state go through the apparatus without the presence of the auxiliary state, recording the output statistics. In this case, the presence of the second beam splitter increases the number of possible output patterns but does not affect the probability to correctly identify a Bell state. Then, the enhanced BSM is tested by switching the ancillary source on and, again, recording the output statistics. We take into account higher-order emission and induced coherence (*46*) by measuring those contributions for each source separately and subtracting those counts from the signal in the postprocessing. Last, the raw count rates are corrected by a factor introduced by the probabilistic photon-number detection (see Methods) to calculate the previously introduced quantities starting from these data.

### Measurement results

**Fig. 3.**
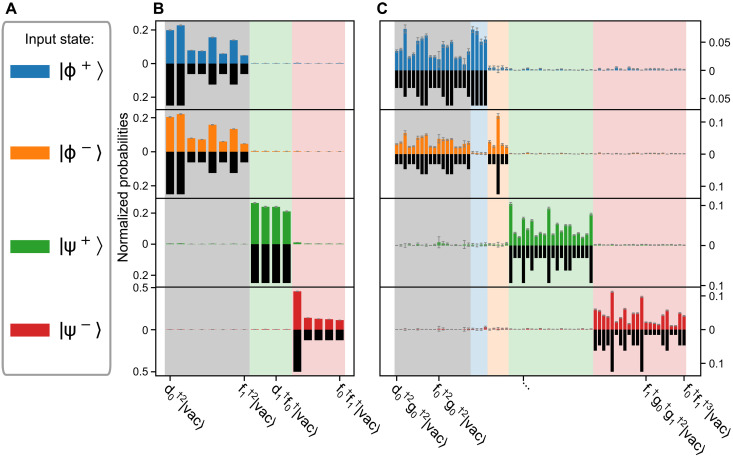
Theoretical detection-probability distributions and measured data. The figure shows the relative probabilities of the detection patterns for each input state (**A**) for standard (**B**) and enhanced (**C**) BSM. BSM. For comparison, theoretical values are represented by black bars and are mirrored with respect to zero. Error bars are shown in gray. The labels represent the photon number for each mode (Eq. 11). While the standard BSM is showing some variations due to experimental imperfections, the measured data are in agreement with the expectations, as captured by the average distance of *D* = 0.072. A standard probability of a correct result of *p*_c_ = 0.481 was achieved. The data of the enhanced BSM also match the theoretical data well, and the enhanced probability has increased to *p*_c_ = 0.579.

We first measure the average probability of the standard BSM. Our results are shown in Fig. 3B. The standard scheme allows us to identify both states ∣Ψ^±^⟩ with over 96% probability, while the states ∣Φ^±^⟩ are completely ambiguous. The measured distributions result in a probability of (48.1 ± 1.2)% to correctly identify the incoming state. As can be seen from Table 1, we achieve high values for parameter MDF (0.9774 ± 0.0002) and low values for the distance *D* (0.0720 ± 0.0049), which indicate the general high quality of optical BSMs. However, our results are close to the theoretical upper bound of 50%, limiting the possibility to increase *p*_c_.

As a next step, we demonstrate the enhanced measurement protocol (see Fig. 3C). While, in the standard BSM, the states |Φ^±^⟩ do not show a unique pattern, the enhanced BSM features a subset of distinguishable outcomes allowing the identification of these states. From the data, we can estimate an average probability of an unambiguous and correct result, with (57.9 ± 1.4)%. In contrast to the previous measurement, we can now identify more than 23% of states ∣Φ^±^⟩, while keeping *p*_c_ for the states |Ψ^±^⟩ above 0.9. This increases the total probability of a correct identification above the theoretical limit of 50%. A full list of characterization parameters for each state is shown in Table 1.

**Table 1.** Measured quantities for both the normal and enhanced method for each of the four Bell states. For the states |Φ^±^⟩, the measurement fidelity is not defined in the normal scheme, since the theory does not allow any unambiguous pattern. N/D, not defined.

Standard method				Enhanced method		
State	*p*_c_	MDF	Distance	*p*_c_	MDF	Distance
|Ψ^+^⟩	0.96 ± 0.02	0.9750 ± 0.0002	0.0646 ± 0.0036	0.906 ± 0.021	0.939 ± 0.011	0.17 ± 0.01
|Ψ^−^⟩	0.966 ± 0.009	0.9797 ± 0.0001	0.0599 ± 0.0012	0.929 ± 0.012	0.943 ± 0.007	0.14 ± 0.01
|Φ^+^⟩	N/D	N/D	0.0789 ± 0.0022	0.248 ± 0.009	0.740 ± 0.022	0.19 ± 0.02
|Φ^−^⟩	N/D	N/D	0.0845 ± 0.0021	0.236 ± 0.009	0.756 ± 0.021	0.17 ± 0.02
Mean	0.481 ± 0.012	0.9774 ± 0.0002	0.0720 ± 0.0049	0.579 ± 0.014	0.845 ± 0.015	0.17 ± 0.02

**Fig. 4.**
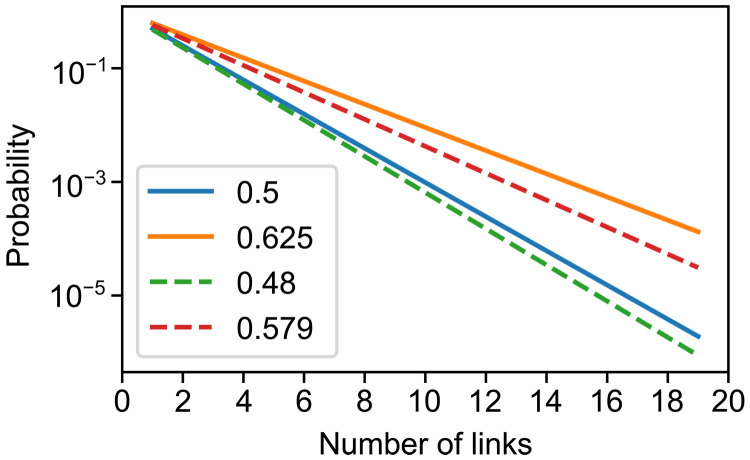
Probability of transmission for a memoryless quantum relay using entanglement swapping. In the figure, different lines represent different BSM success probabilities. Solid lines indicate theoretical values, while dashed lines indicate the experimental values obtained in this work for the standard and the enhanced BSM schemes, respectively.

In Fig. 4, we illustrate the implications of improving the BSM success probability from 50 to 57.9% for a quantum relay that connects the segments of a quantum channel via entanglement swapping at intermediate stations. Such a BSM-based relay achieves, for instance, long-distance privacy. Because of the exponential scaling of the overall success probability with the number of segments *n*, $p_{c}^{n-1}$, already a slightly enhanced swapping efficiency has already a notable impact. While this does not improve the photon-loss scaling in a real fiber channel compared with a point-to-point fiber link, detecting the presence of the photons at the intermediate stations can be beneficial with dark counts. Note that errors in the BSMs, as expressed by our fidelity MDF, would result in a similar scaling with a trade-off between success and error probabilities (both affecting the final secret key rates in quantum key distribution) when considering different types of BSMs. Further note that when the stations are equipped with quantum memories in a quantum repeater chain, the loss scaling is typically improved to $p_{c}^{\log_{2}(n)}$ in certain regimes and settings, and errors could be suppressed by additional rounds of entanglement distillation.

More specifically, reaching links with *n* = 20 like in Fig. 4 will require quantum error detection or correction, independent of the BSM scheme. As an example, for *n* = 8 links, depolarizing error probabilities (“white noise”) smaller than 4% for the BSM at each repeater station are needed to obtain a nonzero secret key rate, assuming standard quantum key distribution protocols and one-way postprocessing (*47*). This is solely due to the imperfect BSMs and independent of the total success probability or additional sources of error (for example, memory imperfections) (*47*). These error rates would correspond to reduced discrimination fidelities, as expressed by (1 − MDF).

Our experiment shows postselected BSM error rates for the enhanced method near 6% and near 25% for the unambiguously identified Bell states ∣Ψ^±^⟩ and |Φ^±^⟩, respectively (see Table 1). This corresponds to an effective, averaged error probability of around 10% for the enhanced scheme. The presented scheme would therefore function in a repeater chain with *n* = 4 segments; however, the secret key rate would be near zero.

In any scalable quantum communication scheme, additional quantum error detection or correction is needed, no matter whether the standard or the enhanced BSM method is used. For the larger systems, however, the impact of the increased efficiency of the enhanced scheme will become more important.

A practical gain can be obtained for an all-photonic memoryless quantum repeater combining many physical qubits into a few logical qubits of a suitable quantum error correction code. For codes being based on single-photon sources and probabilistic linear-optics fusion gates using BSMs, the code’s state generation can be made much more efficient. In particular, the enhancement we achieved in our experimental scheme of 58% compared to the standard 50% leads to a substantial reduction of the overhead.

Let us consider a fault-tolerant long-distance linear-optical quantum communication scheme as described in (*48*). We assume a memoryless repeater every 2 km over a total distance of 1000 km. The scheme is based on logical qubits being encoded in *n* blocks each containing *m* photons. When using a small code, i.e., four blocks with two photons per block, the total average number of photons per node is reduced by a factor of 2 for a BSM success probability of 58% compared to 50%. For a larger code, i.e., 67 blocks with 11 photons per block, we obtain already a reduction of a factor of 5 per node. In total, this allows achieving long-distance distribution success probabilities greater than 50% with a substantial reduction of the overhead. Further improvement to 62.5% or even 75% in the BSM success probabilities would reduce the overhead by even one or even several orders of magnitude, respectively. Note that this quantum communication scheme is partially fault-tolerant (*48*), tolerating depolarizing noise (as it originates in our experiment from multiphoton errors and imperfect mode matching) when the optical states are processed. However, addressing complete fault tolerance, including faulty-state generations related to the finite MDF in our experimental demonstration, would require further theoretical work. The considerable impact of the enhanced BSM on the state generation, and thus also on the total rates, of an all-photonic quantum repeater based on photonic cluster states (*27*), leading to an overhead reduction of several orders of magnitude, was also found in (*29*).

## DISCUSSION

We report the first implementation of a BSM on photonic qubits with a success probability surpassing the 50% limit using only linear optics and two ancillary photons. In our experiments, we reach a success probability *p*_c_ = 57.9%, while a standard BSM, measured under the same experimental conditions, only achieved *p*_c_ = 48%. For this class of BSMs, the two ancillary photons represent the minimal extra resource required to beat that notorious 50% bound, i.e., there is evidence that adding just a single ancillary photon is insufficient (*41*).

We note that our implementation does not require a deterministic auxiliary-state source; even when the source fails to produce a state, BSMs with a 50% success rate are still possible with this setup. This is due to the underlying nature of the scheme to only increase and never reduce any given success probability.

Looking ahead, even higher success rates could be achieved by extending the measurement scheme: The addition of a second ancillary photon pair or ∣Φ^−^⟩ as an auxiliary state would, in principle, boost the maximum success rate to 75%. By further scaling up the setup and adding more ancillary states, the success rate of the measurement can be enhanced arbitrarily close to 100% as shown in (*39*, *40*).

Our results demonstrate how ancillary photon states and linear-optical setups can improve the success rates of BSMs, offering a viable option to boost the general efficiency of any quantum protocol using BSMs. One potential future application could be the creation of large cluster states for measurement-based quantum computing, as it would be possible, applying concepts of percolation theory, to create cluster states of the required size, once the 62.5% efficiency threshold is surpassed with the proper resource states (*20*, *49*). Combined with the significance of BSM efficiency for the field of quantum communication in particular, this work could serve as a stepping stone toward larger quantum networks and more efficient communication links in the future.

## METHODS

### Complete output states

The output states in the modes *f* and *g* are listed below. In case of the states ∣Φ^±^⟩, only a subset of terms is listed: If the number of photons in the mode *d* is not zero, then the state does not interfere with the ancillary state. Therefore, the term must be ambiguous and is not shown here. We only consider the states ∣Ψ^+^⟩ and ∣Φ^±^⟩ as input, since the total photon number in both modes is four. The state ∣Ψ^−^⟩ has only three photons in these two modes and can therefore always be identified by its photon number

$$\begin{array}{c} |\Psi^{-}\rangle |\mathrm{Aux}\rangle \to \frac{1}{8}(-a_{f0}^{†3}a_{f1}^{†}+ia_{f0}^{†2}a_{f1}^{†}a_{g0}^{†}-a_{f0}^{†}a_{f1}^{†}a_{g0}^{†2}+ia_{f1}^{†}a_{g0}^{†3}-ia_{f0}^{†3}a_{g1}^{†}\\ -a_{f0}^{†2}a_{g0}^{†}a_{g1}^{†}-ia_{f0}^{†}a_{g0}^{†2}a_{g1}^{†}-a_{g0}^{†3}a_{g1}^{†}-a_{f0}^{†}a_{f1}^{†3}\\ +ia_{g0}^{†}a_{f1}^{†2}a_{g1}^{†}-a_{f0}^{†}a_{f1}^{†}a_{g1}^{†2}+ia_{f0}^{†}a_{g1}^{†3}-ia_{f1}^{†3}a_{g0}^{†}-a_{f1}^{†2}a_{g0}^{†}a_{g1}^{†}\\ -ia_{f1}^{†}a_{g0}^{†}a_{g1}^{†2}-a_{g0}^{†}a_{g1}^{†3})|vac\rangle \end{array}$$

$$\begin{array}{c} |\Phi^{-}\rangle |\mathrm{Aux}\rangle \to \frac{1}{8}[-\frac{1}{2}\left(a_{f0}^{†4}+a_{f1}^{†4}+a_{g0}^{†4}+a_{g1}^{†4}\right)-a_{f0}^{†2}a_{g0}^{†2}-a_{f1}^{†2}a_{g1}^{†2}\\ -a_{f0}^{†2}a_{f1}^{†2}+a_{f0}^{†2}a_{g1}^{†2}+a_{f1}^{†2}a_{g0}^{†2}-a_{g0}^{†2}a_{g1}^{†2}-4a_{f0}^{†}a_{f1}^{†}a_{g0}^{†}a_{g1}^{†}]|vac\rangle \end{array}$$

$$\begin{array}{c} |\Phi^{+}\rangle |\mathrm{Aux}\rangle \to \frac{1}{8}\left[-\frac{1}{2}\left(a_{f0}^{†4}+a_{f1}^{†4}+a_{g0}^{†4}+a_{g1}^{†4}\right)-a_{f0}^{†2}a_{g0}^{†2}-a_{f1}^{†2}a_{g1}^{†2}\right]|vac\rangle \\ +\frac{i}{4}\left(a_{f0}^{†2}a_{f1}^{†}a_{g1}^{†}-a_{f0}^{†}a_{f1}^{†2}a_{g0}^{†}+a_{f0}^{†}a_{g0}^{†}a_{g1}^{†2}-a_{f1}^{†}a_{g0}^{†2}a_{g1}^{†}\right)|vac\rangle \end{array}$$

The shared terms of the states ∣Φ^±^⟩ are written in bold. The subscript indicates the output mode of the beam splitter. To increase the readability, a full list of all states is shown in Table 2.

**Table 2.** Complete list of possible output states. The table shows the complete list of four-photon states as shown in Fig. 3C, ordered from left to right.

Number	State	Number	State	Number	State
1	$a_{g0}^{†2}a_{d0}^{†2}$ ∣*vac*⟩	24	$a_{f0}^{†2}a_{g1}^{†2}$ ∣*vac*⟩	47	$a_{f1}^{†3}a_{d0}^{†}$ ∣*vac*⟩
2	$a_{g0}^{†2}a_{d1}^{†2}$ ∣*vac*⟩	25	$a_{f1}^{†}a_{f0}^{†}a_{g1}^{†}a_{g0}^{†}$ ∣*vac*⟩	48	$a_{g0}^{†2}a_{d1}^{†}a_{d0}^{†}$ ∣*vac*⟩
3	$a_{g0}^{†4}$ ∣*vac*⟩	26	$a_{f1}^{†2}a_{g0}^{†2}$ ∣*vac*⟩	49	$a_{g0}^{†}a_{g1}^{†3}$ ∣*vac*⟩
4	$a_{g1}^{†2}a_{d0}^{†2}$ ∣*vac*⟩	27	$a_{f1}^{†2}a_{f0}^{†2}$ ∣*vac*⟩	50	$a_{g1}^{†2}a_{d1}^{†}a_{d0}^{†}$ ∣*vac*⟩
5	$a_{f1}^{†2}a_{d1}^{†2}$ ∣*vac*⟩	28	$a_{g0}^{†3}a_{d1}^{†}$ ∣*vac*⟩	51	$a_{g1}^{†3}a_{g0}^{†}$ ∣*vac*⟩
6	$a_{g1}^{†4}$ ∣*vac*⟩	29	$a_{g1}^{†}a_{g0}^{†2}a_{d0}^{†}$ ∣*vac*⟩	52	$a_{f0}^{†}a_{g0}^{†}a_{d1}^{†}a_{d0}^{†}$ ∣*vac*⟩
7	$a_{f0}^{†}a_{g0}^{†}a_{d0}^{†2}$ ∣*vac*⟩	30	$a_{g1}^{†2}a_{g0}^{†}a_{d1}^{†}$ ∣*vac*⟩	53	$a_{f0}^{†}a_{g1}^{†}a_{g0}^{†2}$ ∣*vac*⟩
8	$a_{f0}^{†}a_{g0}^{†}a_{d1}^{†2}$ ∣*vac*⟩	31	$a_{g1}^{†3}a_{d0}^{†}$ ∣*vac*⟩	54	$a_{f0}^{†}a_{g1}^{†3}$ ∣*vac*⟩
9	$a_{f0}^{†2}a_{d0}^{†2}$ ∣*vac*⟩	32	$a_{f0}^{†}a_{g0}^{†2}a_{d1}^{†}$ ∣*vac*⟩	55	$a_{f0}^{†2}a_{d1}^{†}a_{d0}^{†}$ ∣*vac*⟩
10	$a_{f0}^{†2}a_{d1}^{†2}$ ∣*vac*⟩	33	$a_{f0}^{†}a_{g1}^{†}a_{g0}^{†}a_{d0}^{†}$ ∣*vac*⟩	56	$a_{f0}^{†2}a_{g1}^{†}a_{g0}^{†}$ ∣*vac*⟩
11	$a_{f0}^{†2}a_{g0}^{†2}$ ∣*vac*⟩	34	$a_{f0}^{†}a_{g1}^{†2}a_{d1}^{†}$ ∣*vac*⟩	57	$a_{f0}^{†3}a_{g1}^{†}$ ∣*vac*⟩
12	$a_{f0}^{†4}$ ∣*vac*⟩	35	$a_{f0}^{†2}a_{g0}^{†}a_{d1}^{†}$ ∣*vac*⟩	58	$a_{f1}^{†}a_{g0}^{†3}$ ∣*vac*⟩
13	$a_{f1}^{†}a_{g1}^{†}a_{d0}^{†2}$ ∣*vac*⟩	36	$a_{f0}^{†2}a_{g1}^{†}a_{d0}^{†}$ ∣*vac*⟩	59	$a_{f1}^{†}a_{g1}^{†}a_{d1}^{†}a_{d0}^{†}$ ∣*vac*⟩
14	$a_{f1}^{†}a_{g1}^{†}a_{d1}^{†2}$ ∣*vac*⟩	37	$a_{f0}^{†3}a_{d1}^{†}$ ∣*vac*⟩	60	$a_{f1}^{†}a_{g1}^{†2}a_{g0}^{†}$ ∣*vac*⟩
15	$a_{f1}^{†2}a_{d0}^{†2}$ ∣*vac*⟩	38	$a_{f1}^{†}a_{g0}^{†2}a_{d0}^{†}$ ∣*vac*⟩	61	$a_{f1}^{†}a_{f0}^{†}a_{g0}^{†2}$ ∣*vac*⟩
16	$a_{f1}^{†2}a_{d1}^{†2}$ ∣*vac*⟩	39	$a_{f1}^{†}a_{g1}^{†}a_{g0}^{†}a_{d1}^{†}$ ∣*vac*⟩	62	$a_{f1}^{†}a_{f0}^{†}a_{g1}^{†2}$ ∣*vac*⟩
17	$a_{f1}^{†2}a_{g1}^{†2}$ ∣*vac*⟩	40	$a_{f1}^{†}a_{g1}^{†2}a_{d0}^{†}$ ∣*vac*⟩	63	$a_{f1}^{†}a_{f0}^{†2}a_{g0}^{†}$ ∣*vac*⟩
18	$a_{f1}^{†4}$ ∣*vac*⟩	41	$a_{f1}^{†}a_{f0}^{†}a_{g0}^{†}a_{d0}^{†}$ ∣*vac*⟩	64	$a_{f1}^{†}a_{f0}^{†3}$ ∣*vac*⟩
19	$a_{f0}^{†}a_{g1}^{†2}a_{g0}^{†}$ ∣*vac*⟩	42	$a_{f1}^{†}a_{f0}^{†}a_{g1}^{†}a_{d1}^{†}$ ∣*vac*⟩	65	$a_{f1}^{†2}a_{d1}^{†}a_{d0}^{†}$ ∣*vac*⟩
20	$a_{f1}^{†}a_{g1}^{†}a_{g0}^{†2}$ ∣*vac*⟩	43	$a_{f1}^{†}a_{f0}^{†2}a_{d0}^{†}$ ∣*vac*⟩	66	$a_{f1}^{†2}a_{g1}^{†}a_{g0}^{†}$ ∣*vac*⟩
21	$a_{f1}^{†}a_{f0}^{†2}a_{g1}^{†}$ ∣*vac*⟩	44	$a_{f1}^{†2}a_{g0}^{†1}a_{d1}^{†}$ ∣*vac*⟩	67	$a_{f1}^{†2}a_{f0}^{†}a_{g1}^{†}$ ∣*vac*⟩
22	$a_{f1}^{†2}a_{f0}^{†}a_{g0}^{†}$ ∣*vac*⟩	45	$a_{f1}^{†2}a_{g1}^{†1}a_{d0}^{†}$ ∣*vac*⟩	68	$a_{f1}^{†3}a_{g0}^{†}$ ∣*vac*⟩
23	$a_{g1}^{†2}a_{g0}^{†2}$ ∣*vac*⟩	46	$a_{f1}^{†2}a_{f0}^{†}a_{d1}^{†}$ ∣*vac*⟩	69	$a_{f1}^{†3}a_{f0}^{†}$ ∣*vac*⟩

### Pseudo-photon-number resolving detectors

The detectors used to measure the photon states are superconducting nanowire single-photon detectors. While these detectors have very high detection efficiencies of more than 90% for photons at 1550 nm and a very low dark count rate of less than 100 cps, the detectors are binary and cannot distinguish between single- and multiphoton events. To reveal the photon numbers, nevertheless, each mode is equally split into eight modes. Each of these modes connects to a superconducting nanowire detector. The number of coincidences between the detectors reveals the photon number of the original mode. This method is biased though, as multiple photons can be routed to the same output by the splitter. These events result in an incorrect photon number and are discarded in the postselection. The probability for a correct measurement therefore depends on the number of detectors *k* and the number of photons *n* and can be written as

$$P(n,k)=\frac{k!}{(k-n)!}\frac{1}{k^{n}}$$

Since this value depends on the photon number, the measured statistics is shifted by this distribution. To correct for this effect, each measurement outcome of the form ∣*n_dH_n_dV_n_fH_n_fV_n_gH_n_gV_*⟩ has to be multiplied by a factor $\frac{1}{P_{\mathrm{PPNR}}}$

$$P_{\mathrm{PPNR}}=\prod_{i=1}^{6}P(n_{i},k)$$

Here, *k* = 8, as each mode is split onto eight detectors.

### Background correction

The emission from a spontaneous parametric down-conversion source is not a true single photon but a two-mode squeezed vacuum. Using two sources with none of them being heralded leads to induced coherence (*50*). Induced coherence means that higher-order emissions from each of the sources interfere with the signal and thus alters the measurement results, i.e., the click pattern. We measure the contributions from higher-order emissions in separate runs and correct our signal for this background. Doing so requires us to randomize the phase between the Bell and auxiliary states, which is done using a set of waveplates that are rotated to certain settings during the measurement (*51*).

### Estimation of measurement uncertainties

We repeat each experiment over 1000 times for each Bell state, each repetition corresponding to an integration time of 18 s. This sums up to 16 hours of measurement time in total per Bell state. Every measurement cycle collects one output statistics for the BSM, as well as one from the background of each source. After all rounds are completed, a total of *N* output statistics are recorded, each containing the number of detection events *c* for all possible output states |Υ⟩ (see Table 2). We can now derive an average detection rate ${\bar{c}}_{\mathrm{BSM}}$, auxiliary background rate, ${\bar{c}}_{{\mathrm{BG}}_{\mathrm{AUX}}}$, and Bell-state background rate ${\bar{c}}_{{\mathrm{BG}}_{\mathrm{BS}}}$, for every state ∣Υ⟩ by calculating

$${\bar{c}}_{|\Upsilon \rangle }=\sum_{n=1}^{N}\frac{c_{n|\Upsilon \rangle }}{N}$$

By subtracting the average background ${\bar{c}}_{\mathrm{BG}}$ from the average count rate, we obtain the average, background-corrected statistics ${\bar{c}}_{\mathrm{corr}}$ containing a count rate ${\bar{c}}_{\Upsilon \mathrm{corr}}$ for every output state.

To obtain the uncertainty of these data points, we assume a Gaussian distribution. The error for each count rate is given by the propagation of the uncertainty of the measured count rate and the uncertainties of the corresponding backgrounds

$$\Delta c_{|\Upsilon \rangle \mathrm{corr}}=\sqrt{\Delta c_{|\Upsilon \rangle B\mathrm{SM}}^{2}+\Delta c_{|\Upsilon \rangle {\mathrm{BG}}_{\mathrm{AUX}}}^{2}+\Delta c_{|\Upsilon \rangle {\mathrm{BG}}_{\mathrm{BS}}}^{2}}$$

Both Δ*c*_∣Υ⟩BSM_ and Δ*c*_∣Υ⟩BG_ are determined by the standard deviation of the respective statistics

$$\Delta c_{|\Upsilon \rangle }=\sqrt{\sum_{n}^{N}\frac{{\left(c_{n|\Upsilon \rangle }-{\bar{c}}_{n|\Upsilon \rangle }\right)}^{2}}{N}}$$

By calculating the uncertainty for every count rate, we obtain an averaged, background-corrected count rate ${\bar{c}}_{|\Upsilon \rangle \mathrm{corr}}\pm \Delta c_{|\Upsilon \rangle \mathrm{corr}}$ for all measurement results. Uncertainties of quantities derived from this statistics are calculated by error propagation.
